# Living with adrenoleukodystrophy: adult patient and caregiver perspectives

**DOI:** 10.1186/s13023-025-04130-3

**Published:** 2026-01-08

**Authors:** Amena Smith Fine, Kathleen O’ Sullivan-Fortin, Kelly Miettunen, Felicity Emerson, Reza Sadjadi, Ali Fatemi, Florian Eichler

**Affiliations:** 1 Moser Center for Leukodystrophies, Department of Neurogenetics, Kennedy Krieger Institute, 707 North Broadway, Baltimore, MD 21205 USA; 2 ALD Connect, 35 Village Road Suite 100 35533, Middleton, MA 01949 USA; 3 Department of Neurology, Massachusetts General Hospital, 55 Fruit Street, Boston, MA 02114 USA; 4 Department of Neurology, Harvard Medical School, 25 Shattuck Street, Boston, MA 02115 USA; 5 School of Medicine, Department of Neurology, Johns Hopkins, 1800 Orleans St, Baltimore, MD 21287 USA

**Keywords:** Adrenoleukodystrophy, Adrenomyeloneuropathy, Disease burden, Patient voice, Quality of life, Symptoms

## Abstract

**Introduction:**

Adrenoleukodystrophy (ALD) is a rare, X-linked disease caused by pathogenic *ABCD1* gene variants, resulting in heterogeneous and debilitating conditions. We report on an Externally-Led Patient-Focused Drug Development (EL-PFDD) meeting involving patients and caregivers, Food and Drug Administration representatives, and physicians to hear the patient’s voice regarding living with adult manifestations of ALD.

**Methods:**

Adult patients with ALD and/or caregivers were invited to the EL-PFDD to discuss the impact of living with ALD and their desires for future treatment/management.

**Results:**

On July 22, 2022, the virtual EL-PFDD meeting took place with 254 individuals, including 153 adult patients with ALD and/or caregivers. Men and women with ALD suffer from many health conditions with top concerns being balance issues (81%), altered gait (67%), and spasticity (67%). Disease impact on daily activities was significant; 69% had issues with walking, playing sports (45%), and sleeping (41%). Overall, 88% of respondents feared their condition worsening, being unable to walk (61%), and developing cerebral ALD (39%). There are no disease-specific treatments for adults; patients used various medications and physical therapies for symptom management, with either ‘very little’ (39% respondents) or ‘somewhat’ (44% respondents) of a response; 10% received no relief with treatments. In the future, patients want to be involved in treatment development and clinical trials.

**Conclusion:**

ALD is a progressive disease that can be life-limiting. There is an urgent need to develop treatments that will either slow, halt, or cure adult manifestations of ALD, and men and women are eager to be involved in studies.

**Supplementary Information:**

The online version contains supplementary material available at 10.1186/s13023-025-04130-3.

## Introduction

Adrenoleukodystrophy (ALD) is a rare, X-linked disease caused by pathogenic variants in the peroxisomal *ABCD1* gene, resulting in the accumulation of very long chain fatty acids in the plasma and tissues, primarily the brain and spinal cord white matter and adrenal cortex. To date, more than 1,240 unique variants have been identified in the *ABCD1* gene but no genotype/phenotype correlation has been determined [1]. The estimated birth incidence of ALD is 1/14,700 [2] but the introduction of newborn screening suggests that the incidence of ALD could be higher [3].

ALD has a highly complex and poorly predictable clinical presentation and disease course [4]. There are, however, three core clinical phenotypes: (1) a slowly progressive myeloneuropathy (adrenomyeloneuropathy [AMN]); (2) a rapidly progressing leukodystrophy (cerebral ALD [cALD]), and (3) primary adrenal insufficiency. cALD is the most rapidly progressing and devastating type of ALD, with patients becoming completely disabled and typically dying within 4 years after the onset of symptoms [2]. cALD is more common in boys, however adult men with ALD can also develop cALD. As ALD is an X-linked disease, males are more severely affected and can develop all three core clinical phenotypes, while women primarily develop myeloneuropathy [5]. The myriad of symptoms for adult patients with ALD include balance issues, limitations with mobility and walking, leg weakness, restless leg syndrome, spastic paraparesis, ataxia, peripheral neuropathy, sphincter incontinence, sexual dysfunction, fatigue, sleep disturbances, chronic pain, and depression [6–8]. 

Currently there is no cure for ALD, and disease-specific treatments are not available for adult manifestations of the disease. In boys, early stage cALD can be stabilized with the Food and Drug Administration (FDA)-approved gene therapy, SKYSONA, or allogenic hematopoietic stem cell transplantation (HSCT). SKYSONA treatment is not approved for adult patients with cALD. The outcomes of HSCT treatment in adult males with cALD are variable, with treatment being more effective in early stage cALD patients with either no or minor myelopathy symptoms [9]. Unfortunately, however, many men with cALD are not eligible for HSCT due to their advanced age and the absence of a matched donor. They may also be deemed poor candidates based on the severity of their AMN symptoms and extent of brain lesions. Early stage clinical studies with leriglitazone in boys and men with cALD are underway, and a first report suggests treatment may arrest disease progression [10]. Adrenal insufficiency, which has a lifetime prevalence of ~80% in men with ALD [11], should be treated with hormone replacement therapy as it can be life-saving [5]. For patients with myeloneuropathy, supportive treatments, such as pain relief and spasmolytics, may be offered [5]. Clinical surveillance of people with ALD is imperative to monitor for the emergence of treatable aspects of ALD [4]. 

Rare diseases are estimated to affect 263–446 million people worldwide and are considered to be an emerging global public health priority [12]. ALD meets the criteria of a rare disease according to both US and EU definitions [13, 14]. Rare diseases are associated with challenges for patients and the medical profession alike including non-specific disease symptoms; the need for many clinician appointments to obtain a diagnosis; a lack of disease awareness; and uncertain treatment pathways and an absence of disease-specific, licensed treatments [15, 16]. 

With this emerging global public health priority, there is an urgent need for stakeholder engagement to improve the management of rare diseases [17]. Disease-specific, Externally-Led Patient-Focused Drug Development (EL-PFDD) meetings provide key stakeholders, such as the FDA, patient advocates, researchers, drug developers, healthcare providers, and other interested parties, an opportunity to hear the patient’s voice regarding living with disease and how they would like to be involved in drug development. ALD Connect is a non-profit organization that brings together patients, families, physicians, scientists, advocates, and industry, with the aim of improving the quality of life for those living with ALD. ALD Connect undertook an EL-PFDD to understand, from the patient and caregiver perspective, the impact of living with adult manifestations of ALD, including clinician and patient/caregiver understanding of the disease, the availability and impact of current treatments offered to adults living with ALD, and future desires for this patient population.

## Methods

### Patient recruitment for the EL-PFDD

Over a six-month period, adult patients with ALD and/or caregivers of adult patients with ALD were recruited for involvement in the EL-PFDD through advertisements in the ALD Connect newsletter (a bimonthly newsletter emailed to subscribers of the ALD Connect organization) between March and July 2022, use of the ALD Connect website (https://aldconnect.org), and through a targeted social media campaign on Facebook and Instagram comprising seven posts requesting participation. In addition, ALD Connect hosted a community webinar on March 29, 2022, to inform ALD community members about the process of the EL-PFDD and invited adult patient and carer participation. Individuals were deemed eligible to participate if they were over the age of 18 years and either had a known diagnosis of ALD or were a caregiver of an adult with ALD. The number of participants included in the EL-PFDD included patients and/or their caregivers from each clinical subgroup of men with AMN, symptomatic women with ALD, and patients with cALD. Caregivers of patients who had died due to complications of ALD were also included.

Requests by patients and carers to be included in the EL-PFDD implied consent to participate. EL-PFDD meeting attendees were divided into three groups of participants – patients with ALD and/or caregivers who could provide a pre-recorded testimony of living with ALD, patients with ALD and/or caregivers who could provide live testimony during the EL-PFDD meeting (referred to as live panelists), and patients with ALD and/or caregivers who could dial into/join the EL-PFDD meeting and provide verbal or written comments during the meeting. Public speaking and coaching support was provided to those patients who were providing pre-recorded testimonies or who were live panelists for the EL-PFDD meeting. This support was provided by both ALD Connect and an expert in EL-PFDD meetings to help patients with ALD and their caregivers familiarize themselves with the types of questions that could be asked, to ensure that they were able to provide maximum input within the short time frame available for each question, to make sure that standardized disease terminology was used, and to enable speakers to feel comfortable discussing personal aspects of the disease. All EL-PFDD participants were invited to answer polling questions.

### EL-PFDD meeting

The EL-PFDD meeting took place on July 22, 2022, as a virtual meeting conducted over Zoom. The meeting comprised two sessions. Session 1 focused on ALD symptoms and daily impacts of living with this disease. Session 2 covered approaches to treatments for ALD. Each session comprised a panel of people with ALD and people who dialed in to the meeting and was centered around discussion questions and real-time online polling on specific topics. EL-PFDD meeting consultants provided the basic format for the polling questions, which were then tailored and made pertinent for this meeting by ALD Connect staff based on their experience with the ALD community. For maximum attendance, the meeting was livestreamed in both English and Spanish. The meeting was recorded and is available at https://aldconnect.org/pfdd/. After the meeting, the online comment submission portal remained open for a further four weeks to capture the opinions of as many patients with ALD and their caregivers as possible.

### Data storage

The meeting video and meeting report are both available through the ALD Connect website (https://aldconnect.org/pfdd/). No other data from this meeting has been stored.

### Data analysis

The data were reviewed and interpreted by a team of specialists including clinicians and members of the ALD Connect charitable organization. Analysis included a review of the meeting transcripts and the online polling questions, with data being populated in an Excel-based data capture file to ensure consistency across all respondents. Analysts used this information to identify themes that arose most commonly for each question. All data was analyzed descriptively. Where available, categorical variables are presented as numbers and percentages.

## Results

### Meeting attendee demographics

Overall, the EL-PFDD was attended by 254 individuals, including 91 people living with ALD (35.8% of attendees); 62 caregivers, family members, and friends (24.4%); 11 healthcare practitioners (4.3%); 41 attendees from the FDA (16.1%); 27 individuals from the pharmaceutical industry (10.6%); 16 attendees from non-profit entities (6.3%); 5 scientists (2%); and 2 consultants (0.8%). Of the ALD attendees (those either living with ALD or caregivers, family members, and friends of patients with ALD, *n* = 153), 57% were patients with ALD, 28% were caregivers of someone living with ALD, and 15% were both living with ALD and were a caregiver. The majority of ALD attendees lived in the US Eastern time zone (45%), with other attendees coming from the US central time zone (20%), US Pacific time zone (18%), Europe (7%), US mountain time zone (4%), Canada (2%), and other regions (4%). Half of ALD attendees were female (50%). The age demographic of ALD attendees was 19–30 years (7% of participants); 31–50 years (45%); 51–70 years (38%); and those older than 71 years (9%).

### The impact of living with ALD

**Fig. 1:**
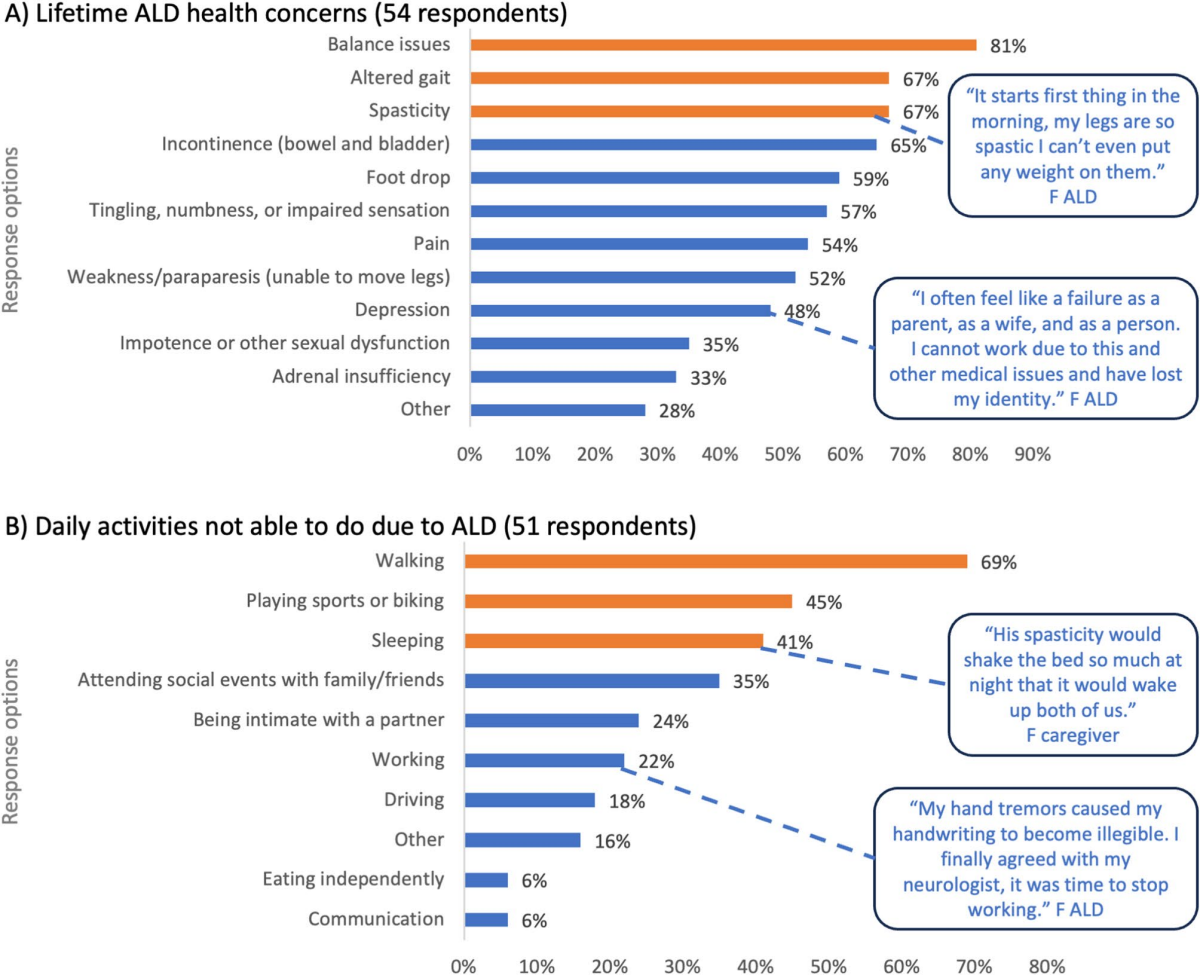
Living with ALD *Legend*: ALD, adrenoleukodystrophy; F, female. The bars on each graph represent the polling question response options. The orange bars represent the top three selected responses and blue bars represent the remaining response options

ALD attendees were invited to take part in a poll to outline their lifetime ALD health concerns, and were allowed to select up to three responses. The responses covered a myriad of health concerns, with the top three responses being balance issues (81% of respondents), altered gait (67%), and spasticity (67%) (Fig. 1A). A 51-year-old symptomatic woman with ALD explained that “*My top challenges right now are gait and balance. And really*,* it starts first thing in the morning when I wake up*,* my legs are so spastic I can’t even put any weight on them*”. More than half of respondents also stated that their lifetime ALD health concerns included bowel/bladder incontinence (65%), foot drop (59%), tingling, numbness, or impaired sensation (57%), pain (52%), and weakness/paraparesis (unable to move legs) (52%). Other responses indicate that 48% of respondents are affected by depression, 35% experience impotence or other sexual dysfunction, and 33% have adrenal insufficiency. 28% of ALD respondents selected the ‘other’ category for health concerns, which included traumatic brain injury from falling, fatigue, severe and repeated infections, and manifestations of cALD including death, seizures, shortness of breath, and short-term memory loss.

In addition to the health concerns associated with ALD, this disease impacts daily activities. In a poll discussing daily activities that are no longer possible due to ALD, the top three responses from ALD respondents were walking (69% of respondents), playing sports or biking (45%), and sleeping (41%) (Fig. 1B). A 43-year-old man living with AMN explained that *“Since the beginning of onset of AMN*,* approximately 15 years ago*,* I have gone from being able to run to using a cane and wheelchair to ambulate daily. I have experienced weekly falls because the coordination*,* muscle atrophy*,* and spasticity in my legs has gotten progressively worse”*. Responses showed that ALD has a significant impact on relationships, with 35% stating they were no longer able to attend social events with family/friends, be intimate with a partner (24%), and communicate effectively (6%). Other responses showed ALD has an impact on typical activities of daily living in adulthood, with respondents reporting they were unable to work (22%), drive (22%), and eat independently (6%).

### Understanding future concerns for people with ALD and their caregivers

**Fig. 2:**
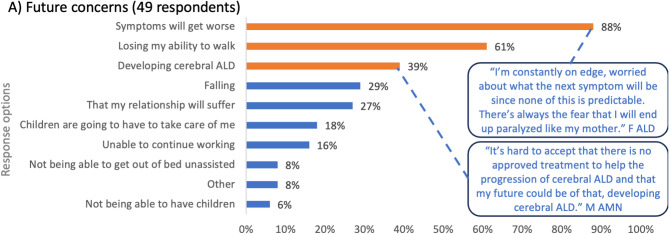
Worries and concerns for the future. *Legend*: ALD, adrenoleukodystrophy; AMN, adrenomyeloneuropathy; F, female; M, male. The bars on the graph represent the polling question response options. The orange bars represent the top three selected responses and blue bars represent the remaining response options

ALD attendees were asked to select their top three concerns for the future. The top three responses were that symptoms would get worse (88% of respondents), losing the ability to walk (61%), and developing cALD (39%) (Fig. 2A). Other concerns included falling, the impact of ALD on relationships, being reliant on family members for care, and not being able to work. Overall, 8% of respondents selected the ‘other’ options, which related to concerns about having to move from their home, having an accident in public, and no longer being able to care for themselves or their loved ones. A symptomatic woman with ALD explained *“I’m constantly on edge*,* worried about what the next symptom will be since none of this is predictable. There’s always fear that I will end up paralyzed the way my mother did. She suffered from ALD for 10 years. Watching her go through that was devastating. I don’t want my family to experience that.”*

### Current treatments for ALD

**Fig. 3:**
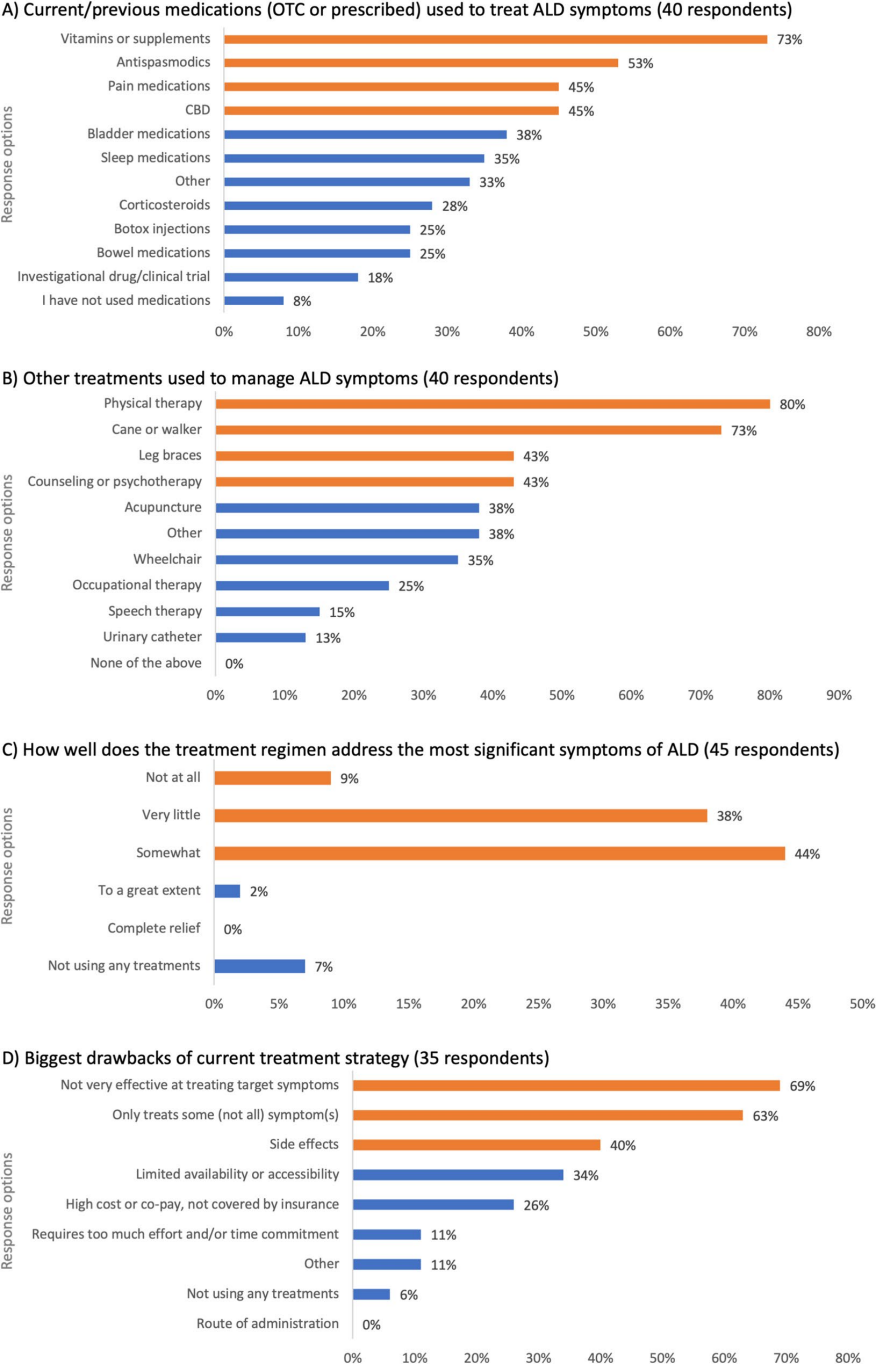
Current treatment for ALD. *Legend*: ALD, adrenoleukodystrophy; CBD, cannabidiol; OTC, over-the-counter. The bars on each graph represent the polling question response options. (**A**) Total number of responses for this graph was 162, meaning each respondent selected an average of 4.2 options. (**B**) Total number of responses for this graph was 160, meaning each respondent selected an average of 4.0 options. The orange bars represent the top three selected responses and blue bars represent the remaining response options

In the absence of an approved treatment specific for adult manifestations of ALD, ALD attendees were asked what medications they had previously used or were using currently to manage their ALD and were asked to select all options that were applicable to their circumstances. Treatment options included both prescribed and over-the-counter medications. The most common treatments were vitamins or supplements (73%), antispasmodics (53%), pain medications (45%), and cannabidiol (CBD; 45%) (Fig. 3A). Other treatments included bladder medications, sleep medications, corticosteroids, Botox injections, bowel medications, and investigational therapies. Only 8% of ALD respondents stated that they had not used medications. For those respondents listing ‘other’ medications, these included bone marrow transplants for cALD, ketamine, off-label medications approved for multiple sclerosis, bladder neck incision surgery, and selective serotonin reuptake inhibitors (SSRIs). One symptomatic woman with ALD and carer of a 21-year-old child with cALD stated that *“I hear something will help and I give it to him … I put* [my son] *on biotin. I heard about vitamin D helping. I put him on vitamin D*,* resveratrol*,* metformin. All these things that I heard are helpful. I went out and got it. And put him on because I don’t want him to miss out.”*

ALD attendees were also invited to share what other non-pharmacologic therapies and aids they use to help manage the symptoms of ALD. Results of the polling showed that interventions included physical therapy (80% of respondents), a cane or walker (73%), leg braces (43%), and counseling or psychotherapy (43%) (Fig. 3B). A symptomatic woman with ALD stated that *“I have to start my day with a series of stretches just to decrease that spasticity. I stretch for about 20 minutes and that allows me to be able to put weight on my legs and take a few steps. As long as I keep moving*,* I’m able to keep my muscles a little bit relaxed.”* Approximately 38% of respondents said they used ‘other’ treatments, which ranged from treatments to address bladder and bowel issues such as pelvic floor treatments and percutaneous tibial nerve stimulation; alternative treatments for spasticity, balance, and walking, including dry needle treatments and electrical stimulation; and personal care aides or modifications to their homes or vehicles. Despite the wide range of treatment regimens used by ALD respondents, they stated that treatments provided either ‘very little’ relief (38%), or ‘somewhat’ relief (44%). Almost 10% of ALD respondents stated that their treatment regimen provided no relief, and there were no respondents who stated that the treatments provided complete relief (Fig. 3C). A man with AMN stated that *“When I have a lot of pain in my legs*,* there is no point in taking medication*,* as they do not contribute to reducing the pain.”*

ALD attendees were asked to explain the drawbacks of their current treatments. The most common responses were that treatments were not very effective at treating target symptoms (69%); treatments only treat some, not all, of their symptoms (63%); and treatments are associated with side effects (40%) (Fig. 3D). 11% of ALD respondents selected the ‘other’ option, citing that responses to treatments are patient-specific; the effect of treatments, such as CBD or Botox, are transient; and that not all patients are eligible for treatments or clinical trials. A symptomatic woman with ALD and caregiver to her adult son with cALD stated that *“There’s nothing for him. He said a million times*,* he’s willing to be a Guinea pig. He wants to help people”*.

### Involvement in clinical trials and treating and managing ALD in the future

ALD respondents have already shown willingness to be involved with novel treatments, with the results of the polling indicating that 18% of respondents had already tried investigational therapies/been involved in clinical trials (Fig. 3A). In testimonials, a symptomatic woman living with ALD stated that *“I’d happily participate in any and all trials available for medications to help”*, while a man living with cALD said *“I was in a blind trial study shortly after being diagnosed*,* and discovered I had the active ingredient. The trial was discontinued. So disappointing!”* and *“Please continue focusing hard on this disease for trials*,* new meds*,* etc. I am sure it is too late for me*,* but am hoping something can be done soon to help those with this disease”*.

**Fig. 4:**
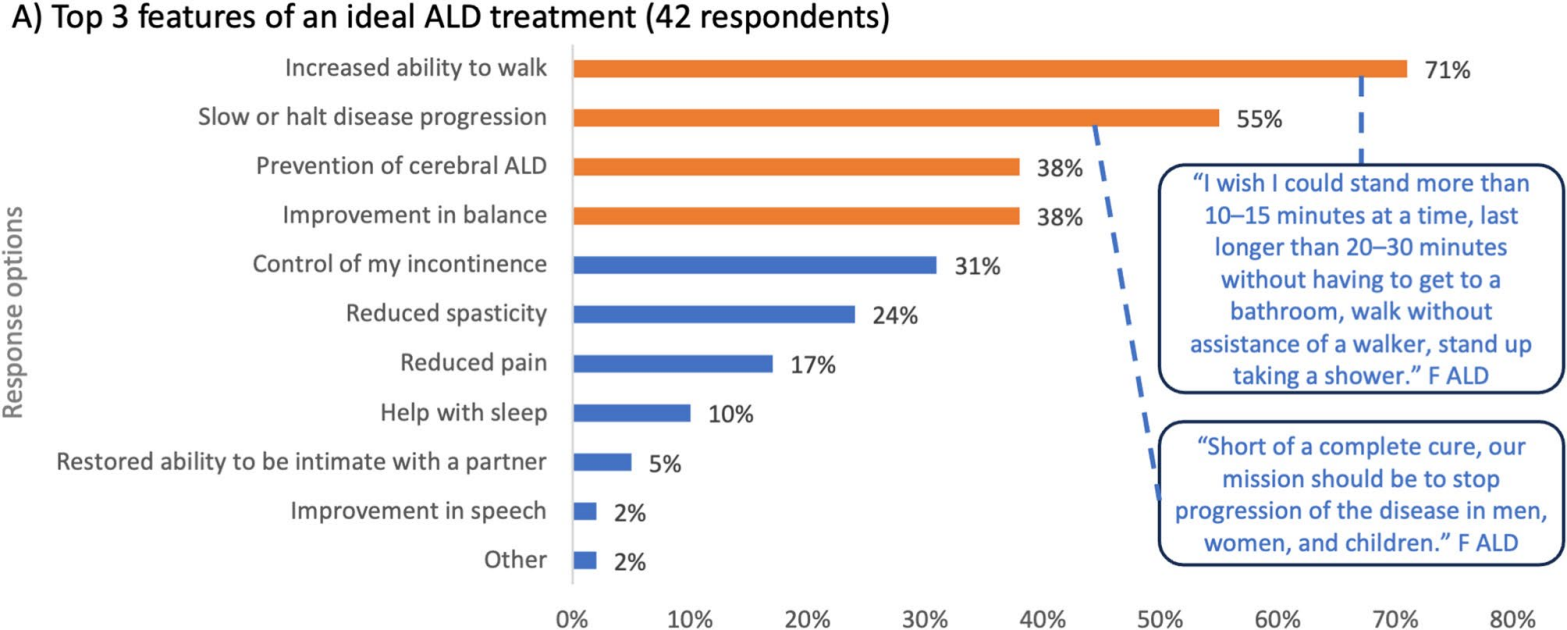
Treating and managing ALD in the future. *Legend*: ALD, adrenoleukodystrophy; F, female. The bars on each graph represent the polling question response options. The orange bars represent the top three selected responses and blue bars represent the remaining response options

Looking to the future, ALD respondents were asked what their top three priorities of a future ALD treatment would include. The key attributes for future treatments were to increase the ability to walk (71%), slow or halt disease progression (55%), prevent the development of cALD (38%), and improve balance (38%) (Fig. 4). For those respondents that selected the ‘other’ option, they hoped that treatments would improve quality of life, lessen fatigue, and improve movement. A man living with AMN explained that *“For treatment*,* I would love it if it could help my walking gait. I really miss a lot of things that I used to do. I used to play basketball. I used to run when I was younger. I had this disease and just some way to regain that would be nice”*.

## Discussion

There are an estimated 7,000 rare diseases, with approximately 80% of rare diseases being attributed to genetics [18, 19] and one of these diseases is ALD [20]. With ALD being a rare disease, there is limited information about its natural history and management – in particular, why the disease has such a heterogeneous presentation, how the disease impacts the lives of patients and their caregivers, which treatments may provide benefit for patients, and what the unmet needs are. The EL-PFDD meeting hosted by ALD Connect was a critical opportunity to showcase the patient voice and highlight the symptoms, impacts, and treatment considerations that are most meaningful to adults living with ALD. The findings from the EL-PFDD show tremendous urgent care needs for all adult patients with ALD.

To date, three core clinical phenotypes of ALD have been described – AMN, cALD, and primary adrenal insufficiency [5]. Men may suffer from all three phenotypes [5], with an 80% lifetime risk of developing adrenal insufficiency and a 20–30% risk of developing cALD in adulthood [11, 21]. Women were originally thought to be asymptomatic carriers of ALD, however, more than 80% of women will eventually develop progressive spinal cord disease and < 1% will develop adrenal insufficiency and cALD [22, 23]. The findings from our polls showed that ALD respondents suffered from a myriad of ALD health concerns that substantially contribute to the overall disease burden irrespective of gender and clinical phenotype. Furthermore, these health concerns meant that there were a significant number of daily activities that these patients were no longer able to do, all of which have a tremendous impact on quality of life. These findings mirror previous studies [6, 8]. The future concerns for patients with ALD included worsening symptoms, losing the ability to walk, developing cALD, falling, impact on relationships, and being unable to continue working. To help alleviate the concern around cALD, more research is needed to understand why some adult men with ALD, irrespective of their genotype/phenotype, are predisposed to developing this life-limiting condition.

People with rare diseases and their caregivers often have mental health challenges that are related to their healthcare, which can be the result of delays in diagnosis, having an uncertain future, lack of or limited treatment options, and facing financial difficulties [24, 25]. The findings of the EL-PFDD are in alignment with other rare diseases and showed that patients with ALD and their caregivers were impacted in a similar way and that ALD is associated with a large psychological burden. One study of X-linked and autosomal recessive diseases showed that women often experienced parental guilt and personal blame for potentially passing a condition to their offspring despite this not being under their control [26]. Parental guilt was also mentioned in the EL-PFDD, with one symptomatic woman with ALD stating that *“When my symptomatic nine-year-old son was diagnosed with adrenoleukodystrophy*,* I was told that I carried the gene as well. When he was on the transplant floor*,* his doctor explained to me the reason for my symptoms was that I was a female with ALD. Guilt*,* knowing I had passed ALD to two of my children*,* and depression revolving around that and knowing my continued loss of abilities would follow me my whole life.”* This highlights an unmet need, where both women and their families need to be provided with appropriate emotional support at diagnosis.

Currently, an estimated 95% of rare diseases do not have an approved treatment [27]. For adult manifestations of ALD, there is neither a cure nor an approved treatment. International recommendations for the diagnosis and management of patients with ALD have been published and include 39 areas of discrete consensus that cover presenting symptoms, the need for multidisciplinary teams to manage patients, screening for cALD, lifestyle management, and the treatment of different symptoms [5]. However, treatment options for the management of ALD symptoms are limited, and the EL-PFDD respondents overwhelming answered that current treatments provided ‘very little’ or only ‘somewhat’ of a response. In the absence of a cure, the EL-PFDD meeting showed that patients with ALD and their caregivers want treatments that improve their quality of life, slow or halt disease progression, and prevent the development of cALD. Furthermore, patients with ALD are willing to be included in clinical trials and, in particular, symptomatic women with ALD would like to be included. Conducting clinical trials in people with rare diseases is challenging due to the small number of patients and potential ethical challenges. As a result, new approaches to determine the effects of treatments in rare diseases are needed including academic-industry collaborations and data sharing to enhance pre-clinical safety studies, the use of modified clinical trials (e.g., individualized studies and removing the placebo groups), using real-world data, using virtual consultations and wearable technology to collect data, and the earlier treatment of diseases (i.e., not waiting until patients are very sick) [28]. Importantly, regardless of the specific approach that is used for the development of novel treatments for ALD, patients with ALD would like to be involved.

Looking to the future for patients with ALD, studies of leriglitazone (a selective peroxisome proliferator-activated receptor subtype gamma agonist) for the treatment of cALD in boys (clinical trial number NCT04528706) and men (NCT03231878, NCT05819866) are currently underway. The ADVANCE study of leriglitazone treatment in adult males did not meet the study primary endpoint of change from baseline in the Six-Minute Walk Test distance at week 96. However, the finding that the development of cALD only occurred in the placebo group suggests that this treatment may slow disease progression in adults with early-stage cALD [29, 30]. In April 2024, SKYSONA™ฏ (elivaldogene autotemcel), an autologous stem cell-based gene therapy, was approved for the treatment of boys aged 4–17 years with early, active cALD without an available human leukocyte antigen (HLA)-matched donor for allogeneic hematopoietic stem cell transplant to slow progression of neurologic dysfunction.[31] While the majority of pharmaceutical development in ALD has focused on mechanisms to treat cALD, treatments to halt or stop the development of AMN are a clear unmet need for both men and women. However, trials targeting AMN have been limited. Recently, a Phase 1/2 gene therapy trial via intrathecal injection had begun recruiting for men with AMN before being discontinued (NCT05394064). Notably, however, symptomatic women with ALD are typically excluded from interventional trials for novel ALD therapeutics. Thus, while further pharmaceutical development is needed for all patients with AMN, a re-evaluation of relevant clinical endpoints and potential inclusion of women is imperative. It is hoped that the findings of this EL-PFDD will drive stakeholders in industry and policymakers to address these unmet needs and to help with guideline development for the treatment of all patients with ALD.

### Study limitations

The biggest challenge for rare diseases is that the disease only affects a minority of the population. While every effort was made to recruit patients with ALD and their caregivers, the number of ALD participants was comparatively small. Another key limitation is that the EL-PFDD meeting was not originally planned as a research study; thus, the key purpose of the meeting was to inform about the symptoms and impacts of ALD in the most effective way, rather than to collect scientific data in a systematic manner. With this in mind, it was not possible to determine from the polls which responses were from male or female patients and which were from caregivers. Of those patients and caregivers attending the meeting, approximately one-third of people responded to the polling questions. Although we cannot be certain of the reasons for this, there are various possible explanations. Some explanations include that responding to the polls was not mandatory, some of the questions or response options might not have been relevant to all attendees, some attendees may not have been able to attend the entire meeting and may have missed some of the polling questions, and the polling questions might have timed out (some attendees stated that there was insufficient time to respond to questions particularly when the questions were longer and had multiple, longer options to choose from). Furthermore, although the EL-PFDD produced a wealth of information, the polling software was limited to 12 responses for each question and, in an ideal scenario, a greater number of options would have been available so more information could have been gleaned. Additional information about the patient experience of ALD care would have also been useful. For example, it would have been interesting to understand the approximate number of clinician appointments that were necessary to obtain a diagnosis, other diagnoses/misdiagnoses received, whether patients were able to attend a center of excellence for their ALD, which clinicians were the medical home for these patients, and whether patients had access to multidisciplinary teams for their care. In addition, the majority of patients involved in the EL-PFDD were adult patients from the US and the findings will be used by the FDA to support medical product development and regulatory decision-making. It would be interesting to hear the patient voice from the caregivers of children with ALD and from other geographical regions, such as Europe and Asia, to determine if these patients with ALD have similar concerns and challenges. Finally, the EL-PFDD focused on ALD from the patients’ perspective but it is also important to give the clinician a voice too, and understand how they manage patients with ALD and what concerns or challenges they face when treating patients.

## Conclusions

The EL-PFDD meeting has provided an in-depth review of the impact of living with ALD on both adult patients and their caregivers, as well as their goals and aspirations for the future management of this disease. ALD is a heterogeneous disease with limited predictable natural history and a lack of data available in the adult population, which significantly impacts the lives of both patients and their caregivers. The most prevalent problems reported by patients were balance problems, altered gait, and spasticity. Daily tasks, like walking, socializing with family and friends, and eating, become difficult and fraught with challenges. Patients have continual concerns about the development of new symptoms and the impact of these on their quality of life. In particular, male patients have concerns and fears of developing cALD, which typically results in death 2–4 years after diagnosis. The debilitating nature of ALD means that male and female patients with ALD have actively requested to be involved and included in future trials of therapies for ALD with the aim of finding disease-specific treatments that will either cure this disease, or slow or halt disease progression.

## Electronic supplementary material

Below is the link to the electronic supplementary material.

Supplementary Material 1

## Data Availability

The EL-PFDD meeting recording and Voice of the Patient Report are available at: https://aldconnect.org/pfdd.
