# Supplementary material for: Living with adrenoleukodystrophy: adult patient and caregiver perspectives
Source: Orphanet J Rare Dis. 2026 Jan 8;21:8. doi: 10.1186/s13023-025-04130-3 (PMC12781523; doi:10.1186/s13023-025-04130-3)
Supplement: Supplementary file 1 — Supplementary Material 1 [file 13023_2025_4130_MOESM1_ESM.docx]

**Appendix I**

**Externally-Led Patient-Focused Drug Development Meeting Agenda**

**July 22, 2022**

**Adrenoleukodystrophy (ALD) in Adulthood**

| 10:00-10:05 AM | **Welcome Remarks**  *Kathleen O’Sullivan-Fortin*  *Symptomatic woman with ALD, ALD Connect Board Member* |
| --- | --- |
| 10:05-10:15 AM | **FDA Introduction to EL-PFDD**  *Wilson Bryan, MD*  *Director, Office of Tissues and Advanced Therapies, FDA* |
| 10:15-10:30 AM | **Clinical Features of ALD and Adrenomyeloneuropathy (AMN) & Therapeutic Approaches**  *Florian Eichler, MD*  *Associate Professor of Neurology at Harvard Medical School, ALD Connect Board Member* |
| 10:30-10:35 AM | **Meeting Overview and Introduction**  *James Valentine, JD, MHS, Meeting Moderator* |
| 10:35-10:40 AM | **Demographic Polling**  *James Valentine, JD, MHS Meeting Moderator* |

| **Session 1: Living with ALD: Men with AMN, cerebral ALD (cALD), symptomatic women with ALD** | |
| --- | --- |
| 10:40-11:05 AM | **Pre-recorded Panelists** |
| 11:05-12:30 PM | **Polling and Audience Discussion** • Zoom Discussion Starters (live) • Patient/caregiver audience remote polling • Moderated audience discussion (telephone and written comments) |
| 12:30-1:00 PM | **Break** |

| **Session 2: Approaches to Treatments for ALD and AMN: Men with AMN, cALD, symptomatic women with ALD** | |
| --- | --- |
| 1:00-1:25 PM | **Pre-recorded Panelists** |
| 1:25-2:40 PM | **Polling and Audience Discussion** • Zoom Discussion Starters (live) • Patient/caregiver audience remote polling • Moderated audience discussion (telephone and written comments) |
| 2:40-2:50 PM | **Meeting Summary**  *Larry Bauer, RN MA* |
| 2:50-2:55 PM | **Concluding Remarks & Next Steps**  *Kathleen O’Sullivan-Fortin*  *Symptomatic woman with ALD, ALD Connect Board Member* |
